# SPNet: Structure preserving network for depth completion

**DOI:** 10.1371/journal.pone.0280886

**Published:** 2023-01-24

**Authors:** Tao Li, Songning Luo, Zhiwei Fan, Qunbing Zhou, Ting Hu

**Affiliations:** School of Electrical Engineering and Electronic Information, Xihua University, Chengdu, China; Wuhan University of Science and Technology, CHINA

## Abstract

Depth completion aims to predict a dense depth map from a sparse one. Benefiting from the powerful ability of convolutional neural networks, recent depth completion methods have achieved remarkable performance. However, it is still a challenging problem to well preserve accurate depth structures, such as tiny structures and object boundaries. To tackle this problem, we propose a structure preserving network (SPNet) in this paper. Firstly, an efficient multi-scale gradient extractor (MSGE) is proposed to extract useful multi-scale gradient images, which contain rich structural information that is helpful in recovering accurate depth. The MSGE is constructed based on the proposed semi-fixed depthwise separable convolution. Meanwhile, we adopt a stable gradient MAE loss (*L*_*GMAE*_) to provide additional depth gradient constrain for better structure reconstruction. Moreover, a multi-level feature fusion module (MFFM) is proposed to adaptively fuse the spatial details from low-level encoder and the semantic information from high-level decoder, which will incorporate more structural details into the depth modality. As demonstrated by experiments on NYUv2 and KITTI datasets, our method outperforms some state-of-the-art methods in terms of both quantitative and quantitative evaluations.

## 1 Introduction

Each pixel value of the depth map represents the range between scene and the camera. Thus, depth maps have been widely used in various computer vision tasks, such as autonomous driving, robot obstacle avoidance and unmanned aerial vehicle control. However, current depth maps captured by consumer depth sensors are usually noisy and extremely sparse. The goal of depth completion is to generate a dense depth map from a sparse one. This task is usually guided by a corresponding high-resolution RGB image.

There are still some challenges in depth completion. **Firstly**, it is difficult to well preserve accurate depth structures, such as tiny structures and object boundaries. Thus, the depth completion results generated by many methods usually suffer from value ambiguity artifacts [1]. **Secondly**, RGB-guided depth completion methods need to consider the problem of the modal distinction. The model distinction between depth maps and RGB images is that their values represent range and intensity respectively.

In order to tackle the depth value ambiguity problem, existing methods have introduced surface normal [2–5], context semantic information [6–9], or uncertainty estimation [10–13] to improve the accuracy of boundary values and preserve more structures. However, most of these methods require extra information or datasets. Different from them, we attempt to capture more useful structural information just from the input RGB images, which contain very rich semantic structures and sharp object boundaries [7–9, 14]. Specifically, we propose a multi-scale gradient extractor (MSGE) to generate gradients from the input RGB images. We firstly scale-wisely down-sample the RGB images followed by a corresponding up-sampling to obtain multi-scale RGB images. Then, by using the proposed semi-fixed depthwise separable convolution, multi-scale gradient features are adaptively learned from these obtained RGB images. The proposed semi-fixed depthwise separable convolution can also be used in flexible semi-fixed learning tasks. Besides, we employ a stable gradient MAE loss (*L*_*GMAE*_) to eliminate geometric distortions and boundary ambiguities. *L*_*GMAE*_ has been found to be more stable and better than the gradient MSE loss through our experiments.

Considering the fact that RGB data modality and depth data modality have different statistical properties, various fusion methods [4, 15–25] have been proposed to eliminate the modal distinction to better fuse RGB and depth information. Jaritz et al. [15] demonstrated that the middle fusion performed better than the early fusion strategy. Thus, we follow the middle fusion strategy and propose a multi-level feature fusion module (MFFM). Different from previous fusion methods, our MFFM considers the modal distinction through the enhancement attention and the complementation attention. The MFFM adaptively combines the detailed spatial information from low-level RGB encoder features with the accurate semantic information from high-level depth decoder features. Therefore, more structural details from RGB modality will be incorporated into the depth modality.

In addition, due to the effectiveness in modeling multi-scale contextual information, Atrous Spatial Pyramid Pooling (ASPP) or its variants have showed promising performance in semantic segmentation [26–28], stereo matching [29–31] and object detection [32–34]. We also add ASPP on the final layer of our SPNet to further enhance the learning capability of our network.

In general, our main contributions are in three aspects:

1. We propose a multi-scale gradient extractor to extract multi-scale gradient images from input RGB images and then feed them into the network to better preserve structure boundaries of depth maps. MSGE is constructed based on the proposed semi-fixed depthwise separable convolution.
2. We adopt a gradient MAE loss to constrain depth gradients, which will make the network pay more attention to geometric structures and object boundaries.
3. A multi-level feature fusion module is introduced to adaptively fuse spatial details and semantic information from low-level encoder and high-level decoder. MFFM comprehensively considers modal distinction, and thus introduces more structural information into depth modality.

## 2 Related work

### 2.1 Depth completion

Deep neural networks greatly promote the development of depth completion task. At present, the related works of depth completion can be roughly divided into three main categories: single-branch-based methods [4, 18, 19, 35–38], two-branch-based methods [16, 17, 20, 22, 39–43] and multiple-branch-based methods [3, 25, 44]. The single-branch-based methods use only one encoder-decoder network to complete depth maps. For example, Chen et al. [18] used one hourglass network to complete depth maps by learning joint 2D-3D representations. Ma et al. [19] fed concatenated RGB and depth features into a single hourglass network and used a self-supervised training framework. The two-branch-based methods input the depth map and RGB image into two hourglass networks, respectively. For example, Tang et al. [16] used two independent encoder-decoder network to extract the RGB and depth features respectively, and then designed a guided convolution module to fuse the decoder features from RGB branch with the encoder features from depth branch. Besides, some multiple-branch-based works [3, 44] employed three hourglass networks to extract more useful information. Yan et al. [44] indicated that the increasing number of hourglass networks could improve the depth completion performance. Although these existing methods do improve the overall quality of depth maps, further research is still necessary to better preserve depth structures.

Some works also exploited additional refinement networks. The most representative one is spatial propagation network [22, 35–38], which propagates information according to the affinities that are learned from fixed [22, 35, 36] or variable [37, 38] kernels. The convolutional spatial propagation network (CSPN) [35] was firstly proposed by Cheng et al. to learn affinity values from local neighbors and then refine the depth completion results. CSPN++ [36] adaptively learned the number of iterations and the size of the convolution kernel to further increase its effectiveness and efficiency. NLSPN [37] utilized relevant information from non-local neighbors and excluded irrelevant neighbors during propagation. DSPN [38] proposed a deformable spatial propagation network to adaptively learn offsets and affinity matrices. PENet [22] introduced a dilated and accelerated CSPN++ to enlarge the neighborhoods and improve inference speed. However, these methods usually have huge inference time and have shown poor generalizability on variable sparsity.

### 2.2 Gradient-related methods

Gradient information has been used in previous depth completion works, such as [45–50]. Commonly, there are two ways to introduce gradient information into deep networks: 1) incorporating gradients into the model to guide depth completion [45], and 2) introducing gradients into the loss for constraints [45–50]. Specifically, Hwang et al. [45] designed a teacher network to learn gradient depth images, which were then used to train their geometrical edge CNN through a Knowledge-Distillation loss function. As a parametric method, their gradient generation method required more computing resources. Nguyen et al. [46] and Ryu et al. [47] adopted a gradient-related loss to encourage local smoothness of depth predictions. Gu et al. [48] used some pseudo depth maps to rectify input sparse depth, and supervised training by some structural losses including gradient loss. Liu et al. [49] applied a constraint on depth gradient to penalize the disagreement of depth boundaries. Hegde et al. [50] proposed a novel Gradient Aware Mean Squared Error Loss (GAMSE) to preserve boundary information in predicted dense depth maps.

Compared to [45], our network introduces an efficient multi-scale gradient extractor, which has a better structure-preserving ability. Specifically, the MSGE can not only retain boundaries of small-size objects, but also eliminate redundant textures inside the large-size objects. Besides, we employ a gradient MAE loss to compute the gradient error between predicted and ground truth depth maps. Since the depth gradient images provide extra structural constraints, the model trained with *L*_*GMAE*_ can better restore geometric structures and enhance boundary sharpness.

### 2.3 Multi-modal fusion

Multi-modal fusion strategy can be roughly divided into three ways: early, middle and late fusion. Specifically, the early fusion [4, 18, 19, 21, 51] simply concatenated two modalities and then directly fed them into the same encoder. This fusion method did not fully consider the distinction of different modalities. The middle fusion [16, 17, 20, 22, 52] fused different modalities on their intermediate features. Late fusion [3, 17, 22] aggregated multiple dense depth maps predicted by different branches. Jaritz et al. [15] demonstrated that the middle fusion had better performance than the early fusion strategy. Most of the existing middle fusion methods only exploited simple concatenation or summation operation, which cannot effectively fuse multi-modal information. Some recent works had explored several effective fusion methods, such as guided convolution module [16], multi-modal masked pre-training (M3PT) [24], adaptive symmetric gated fusion [17] and channel shuffle [20]. Although PENet [22] considered both the early and late fusion, they only used simple concatenate operations. Different from these works, we propose a multi-level feature fusion module to exploit the cross-level information between low-level encoder features and high-level decoder features. The MFFM fully considers the modal distinction and thus more useful structure details from RGB modality will be incorporated into the depth modality.

## 3 Methodology

**Fig 1:**
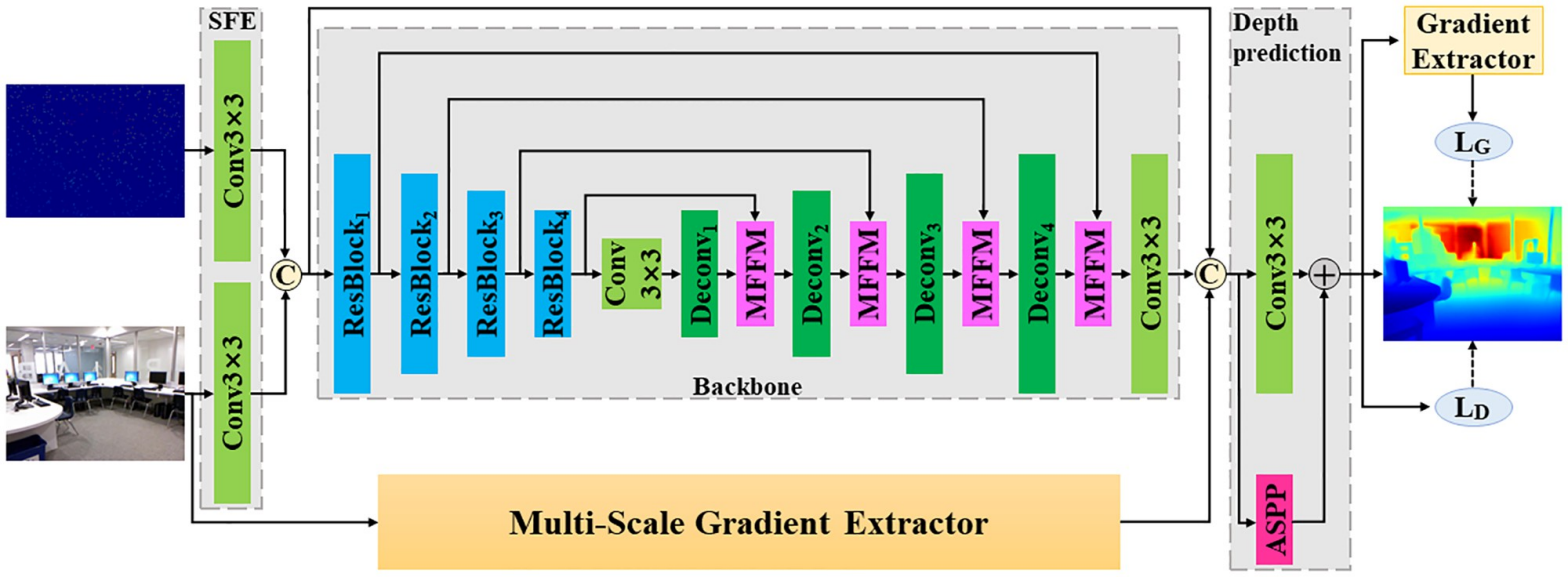
The overall architecture of the proposed SPNet. SFE represents shallow feature extractor. MFFM denotes the proposed multi-level feature fusion module. *L*_*D*_ and *L*_*G*_ represent the depth loss and gradient MAE loss (*L*_*GMAE*_), respectively.

We design an end-to-end structure preserving network (SPNet) for depth completion. SPNet mainly contains four parts: shallow feature extraction (SFE), UNet backbone, multi-scale gradient extractor, and depth prediction, as shown in Fig 1.

Sparse depth maps and RGB images are fed into two shallow feature extractors, respectively. And then the depth shallow features and the color shallow features are concatenated and fed to the backbone. Our backbone is an UNet with an encoder and a decoder. The encoder consists of four residual layers and a convolutional layer. The decoder consists of four deconvolution layers, four MFFMs and one convolutional layer. We use MFFMs to adaptively fuse the low-level encoder features and the high-level decoder features, instead of simply concatenating or summing them.

The MSGE extracts multi-scale gradients from RGB images and then adaptively fuses them. Then, the fused gradient features from MSGE, the depth features from backbone, and the concatenated shallow features are combined into a subsequent concatenation layer. And we find that a late concatenation of gradient features and depth features have a high efficiency than an early concatenation. Finally, the concatenated features are fed to the depth prediction module, which is composed of a convolutional layer and an ASPP layer.

Our network will be optimized with a loss function based on a depth constraints *L*_*D*_ and a gradient constraints *L*_*G*_. In *L*_*G*_, we use a Sobel detector to extract gradients from depth maps.

### 3.1 Multi-scale gradient extractor (MSGE)

**Fig 2:**
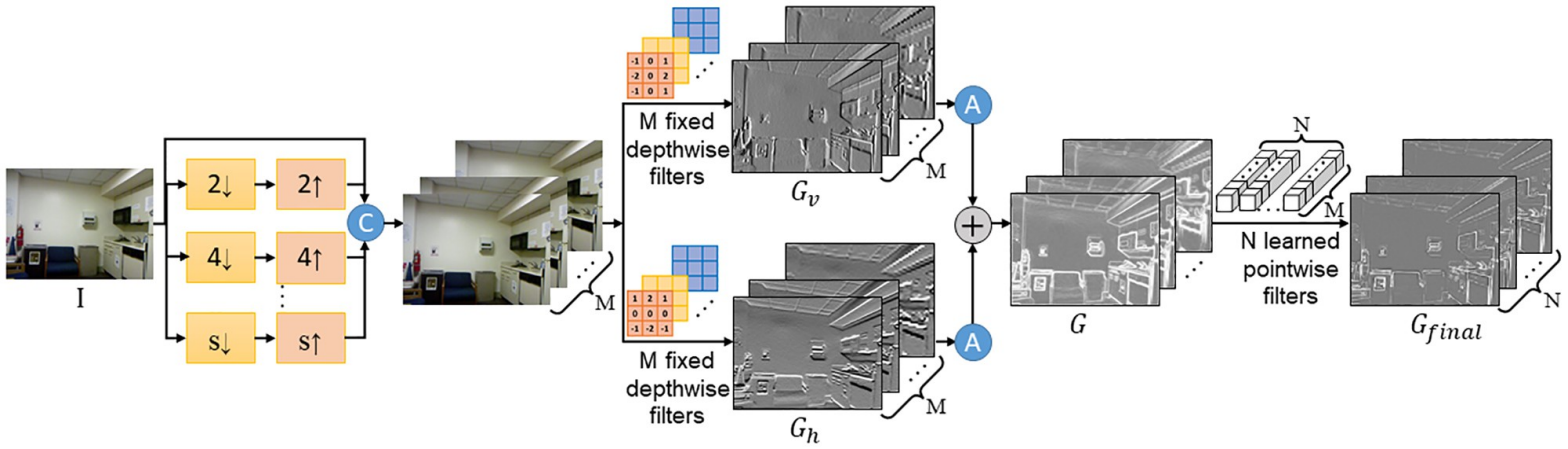
The architecture of the multi-scale gradient extractor.

The multi-scale gradient extractor is used to extract gradient features from input RGB images. As we know, RGB images contain sharp boundaries and rich textures, and their gradients correspond to the high-frequency details, which can improve the depth completion performance. It is not difficult to extract gradient images from RGB images. However, how to extract useful gradients for depth completion is still a challenging problem, because the gradient images that directly extracted from RGB images contain a lot of redundant texture information. These redundant textures may bring explicit texture transferring artifacts. Thus, we design an efficient gradient extractor, which can provide rich boundary structures, as well as restrain redundant textures. The architecture of this extractor is shown in Fig 2.

Firstly, we down-sample the RGB images *I* by a scale factor *s*, and then we use bicubic interpolation to up-sample them to the original size. The scale factor *s* is in a scale set *S*. In our experiment, set *S* is {1, 2, 4}. In this way, we obtain multi-scale RGB images.

**Fig 3:**
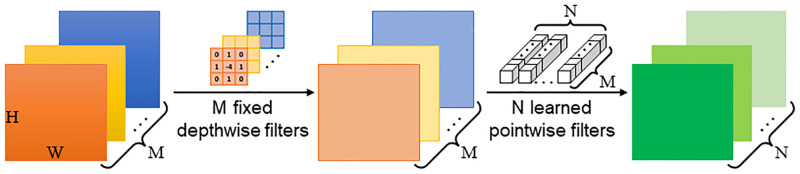
The proposed semi-fixed depthwise separable convolution.

Then, these multi-scale RGB images are concatenated and fed to the proposed semi-fixed depthwise separable convolution, as shown in Fig 3. As we all know, a depthwise separable convolution [53] consists of two parts: a depthwise convolution and a pointwise convolution. The proposed semi-fixed depthwise separable convolution fixes the depthwise convolution with given kernel weights, while keeps the pointwise convolution learnable. The proposed semi-fixed depthwise separable convolution can also be applied to other cases of semi-fixed learning.

The filter number of the 2D depthwise convolution is set to *M*, which equals to the channel number of the concatenated multi-scale RGB images. We choose vertical and horizontal Sobel filters as the fixed depthwise filters of each channel to obtain the multi-scale vertical boundaries *G*_*v*_ and multi-scale horizontal boundaries *G*_*h*_, respectively. This process can be formulated as:

$$\begin{array}{c} G_{v}=Sobel_{v}([I||\cdots ||(up_{s}(down_{s}\left(I\right)))]) \end{array}$$ (1)

The horizontal boundaries *G*_*h*_ can be obtained in the same way. Then, we can obtain the multi-scale gradient images *G* by combining the vertical and horizontal boundaries through the following formula:

$$\begin{array}{c} G=A\left(G_{v}\right)+A\left(G_{h}\right), \end{array}$$ (2)

where *A*(⋅) represents the absolute value function.

There are several advantages of fusing multi-scale gradient images. Specifically, the small-scale gradient images extracted from small-scale RGB images contain rich boundary information of small-size objects as well as redundant textures, while the large-scale gradient images can eliminate the redundant texture inside large-size objects. Thus, to make full use of different scale gradient images and adaptively fuse them, we feed these multi-scale gradient images *G* to the pointwise convolution that with *N* learnable pointwise filters to obtain the final gradient images *G*_*final*_. The *G*_*final*_ are then integrated into the model to provide additional structural information for the high-level depth features.

### 3.2 Multi-level feature fusion module (MFFM)

**Fig 4:**
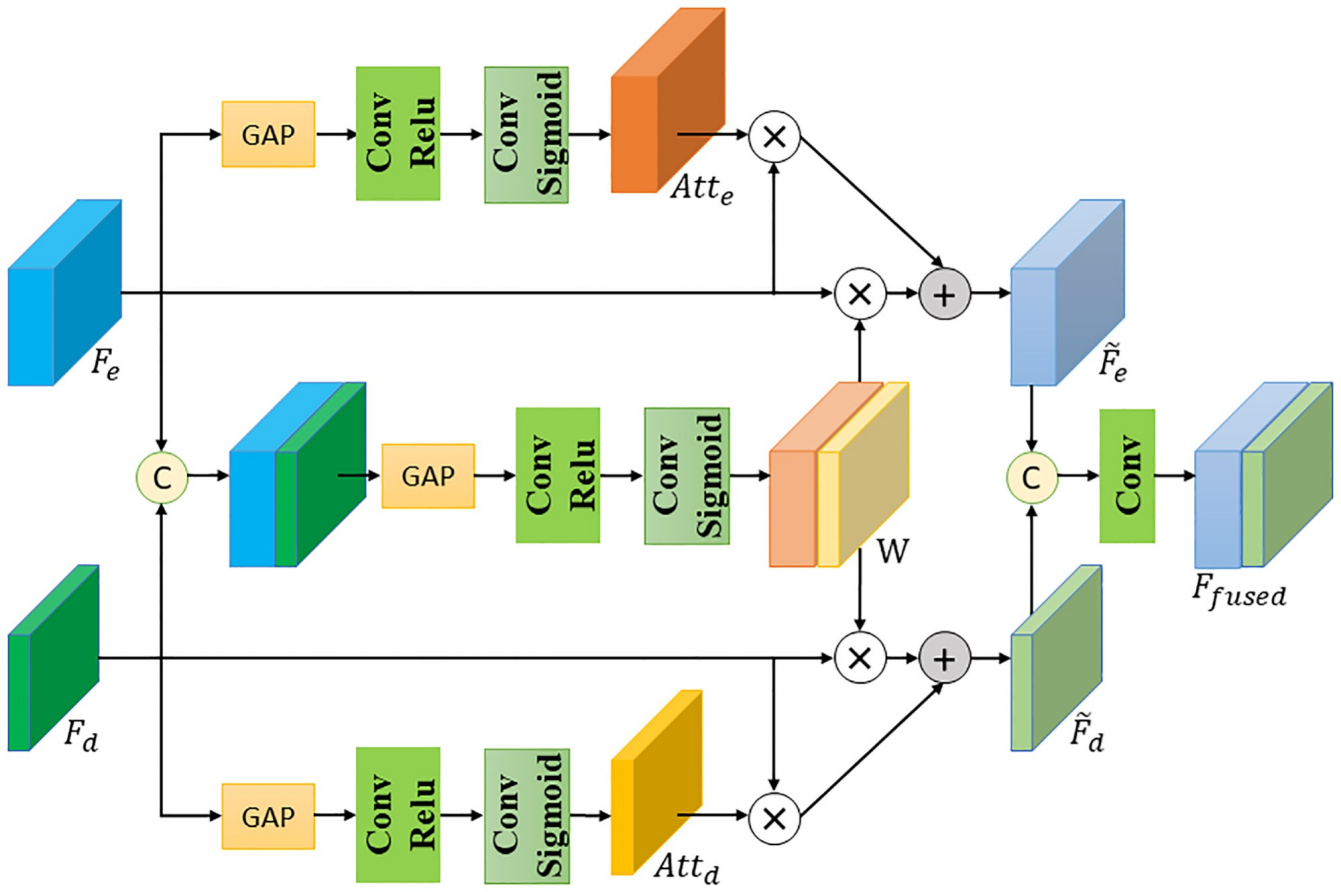
The architecture of the proposed multi-level feature fusion module. The GAP denotes the global average pooling operation. The *Conv* in MFFM is all 1×1 convolutional layer.

In order to adaptively fuse the spatial details from low-level encoder and the semantic information from high-level decoder, we propose a multi-level feature fusion module and use it in a middle fusion strategy. As shown in Fig 4, the processing stage of the proposed MFFM can be roughly divided into: information enhancement stage, information complementation stage, and multi-level feature fusion stage. MFFM will generate features *F*_*fused*_ by fusing the low-level encoder features *F*_*e*_ and the high-level decoder features *F*_*d*_.

In the information enhancement stage, we take a global average pooling (GAP) to compress features channel-wisely. This GAP operation can capture the spatial detail information from *F*_*e*_ and the global semantic and structural information from *F*_*d*_. Then, these two features are separately fed into two 1×1 convolutional layers to learn channel-wise fusion weights. Finally, a sigmoid activation function *σ*(⋅) is adopted to normalize these weights. The enhancement attention *Att*_*e*_ for encoder features can be adaptively obtained as:

$$\begin{array}{c} Att_{e}=\sigma (Conv(GAP(F_{e}))), \end{array}$$ (3)

where *Conv*(⋅) and *GAP*(⋅) represents the functions of these two convolutional layers and the global average pooling, respectively. The enhancement attention *Att*_*d*_ for decoder features can be obtained in the same way.

In the information complementation stage, we concatenate the input encoder features *F*_*e*_ and decoder features *F*_*d*_. In a similar way as the enhancement stage, a GAP/Conv combination followed by a sigmoid activation is applied on the concatenated features to adaptively obtain a joint weight *W*. *W* represents the correlation and complementarity between cross-level features *F*_*e*_ and *F*_*d*_. The complementation attention *W* can be obtained as:

$$\begin{array}{c} W=\sigma (Conv(GAP([F_{e}\left|\right|F_{d}]))), \end{array}$$ (4)

where [⋅||⋅] denotes the concatenation operation.

Then, we rescale features by using the learned enhancement attention *Att* and the complementation attention *W*, and the re-weighted encoder features ${\tilde{F}}_{e}$ and the re-weighted decoder features ${\tilde{F}}_{d}$ can be formulated as:

$$\begin{array}{c} {\tilde{F}}_{e}=F_{e}⊗Att_{e}+F_{e}⊗W\left[0:c_{e}\right], \end{array}$$ (5)

$$\begin{array}{c} {\tilde{F}}_{d}=F_{d}⊗Att_{d}+F_{d}⊗W\left[c_{e}:c_{e}+c_{d}\right], \end{array}$$ (6)

where *c*_*e*_ and *c*_*d*_ represent the channel number of *F*_*e*_ and *F*_*d*_, respectively.

In the final multi-level feature fusion stage, ${\tilde{F}}_{e}$ and ${\tilde{F}}_{d}$ are concatenated and fed to a 1×1 convolutional layer to generate the final fused features *F*_*fused*_:

$$\begin{array}{c} F_{fused}=Conv_{1\times 1}([{\tilde{F}}_{e}||{\tilde{F}}_{d}]) \end{array}$$ (7)

Considering the modality distinction, the proposed MFFM enhances intrinsic modal information through the enhancement attention. It also balances complementary modal information through the complementation attention. Thus, by combining the enhancement attention weight and the complementation weight, more discriminative spatial information from low-level features and more accurate semantic information from high-level depth decoder are learned to better preserve object structures.

### 3.3 Loss function

#### Overall loss function

For training our network, the overall loss function L is defined as a weighted sum of a conventional depth loss *L*_*D*_ and a gradient loss *L*_*G*_:

$$\begin{array}{c} L=L_{D}+\lambda L_{G} \end{array}$$ (8)

#### Depth loss

Mean absolute error (MAE) (i.e. *l*_1_ loss) and mean squared error (MSE) (i.e. *l*_2_ loss) are two commonly used loss function in depth completion. In our experiments, we train our network by using *l*_1_ and *l*_2_ simultaneously. Following previous works, we only consider pixels with valid depth values when computing the loss. This loss function can be defined as:

$$\begin{array}{c} L_{D}=\frac{1}{N}\sum_{q\in Q}|D_{q}^{gt}-D_{q}^{pred}|+\frac{1}{N}\sum_{q\in Q}{\left(D_{q}^{gt}-D_{q}^{pred}\right)}^{2}, \end{array}$$ (9)

where Q refers to the set of valid pixels in GT images, and N is the number of pixels in Q.

#### Gradient MAE loss

To eliminate geometric distortions and boundary ambiguities, we adopt a gradient MAE loss *L*_*G*_, which computes the MAE error between the gradient images of the completion results and the gradient images of the GT depth:

$$\begin{array}{c} L_{G}=\frac{1}{N}\sum_{q\in Q}|G(D_{q}^{gt})-G(D_{q}^{pred})|, \end{array}$$ (10)

where *G*(⋅) represents the Sobel gradient detector. The *L*_*GMAE*_ encourages consistency of depth gradients, which can make the model pay more attention to geometric structures and object boundaries.

## 4 Experiments

In order to evaluate the effectiveness of the proposed network, we conduct experiments on two benchmark datasets: NYU Depth V2 dataset [54] (NYUv2) and KITTI Depth Completion dataset [55] (KITTI DC). In this section, we firstly summarize the implementation details: parameter setting and evaluation metrics. Then, we compare the proposed model with the state-of-the-art methods quantitatively and qualitatively. Finally, we perform ablation experiments to verify our network components, including multi-scale gradient extractor, gradient MAE loss and multi-level feature fusion module.

### 4.1 Implementation details

#### Parameter setting

Our SPNet is implemented in PyTorch and trained on two NVIDIA 2080Ti GPUs. In specific, we optimize the proposed model by using ADAM [56] optimizer with *β*_1_ = 0.9, *β*_2_ = 0.999 and weight decay *ϵ* = 1*e*^−6^. Our network is trained end-to-end for 20 epochs with a batch size of 18. The initial learning rate is set to 1*e*^−3^ and decayed by {1/1, 1/5, 1/25} at epoch {10, 15, 20}. The scale factor *s* in MSGE is set to {1, 2, 4}. The filter number *M* of the 2D depthwise convolution is set to 9, and the filter number *N* of the learnable pointwise convolution is set to 3. The hyperparameter λ in loss function is set to 1 in our model.

#### Evaluation metrics

For indoor NYUv2 dataset, the quantitative performance is evaluated in terms of root mean square error (RMSE in meter), mean absolute relative error (REL in meter), and *δ*_*i*_ which denotes the percentage of relative errors inside a certain threshold *i* (*i* ∈ 1.25, 1.25^2^, 1.25^3^). For outdoor KITTI DC dataset, four commonly used metrics are used to evaluate quantitative performance: root mean squared error (RMSE), mean absolute error (MAE), root mean squared error of the inverse depth (iRMSE), and mean absolute error of the inverse depth (iMAE). RMSE and MAE are in millimeters (mm), while iRMSE and iMAE are in 1/kilometers (1/km). RMSE is used as the primary metric on these two datasets in our experiments.

### 4.2 Comparisons with state-of-the-arts on NYUv2 dataset

#### NYUv2 dataset

The NYUv2 dataset [54] consists of indoor dense depth maps and corresponding RGB images captured by Microsoft Kinect. These images are collected from 464 indoor scenes, of which 249 scenes are used for training and another 215 scenes for testing. Following previous works [3, 57], our model is trained on 50K frames out of the training set, and tested on the official labeled test set with 654 images. The original images of size 640×480 are firstly down-sampled to 320×240, and then center-cropped to size 304×228. The sparse depth maps are obtained by randomly sampling 500 points from the dense depth maps.

#### Quantitative comparison

We first evaluate our method quantitatively on the 654 test samples of the NYUv2. The quantitative results are reported in Table 1. In general, geometry-related methods [1, 3, 4, 58, 59] and fusion-related methods [16, 17, 20] perform better than S2D [21] and NConv-CNN [60]. The general superiority of the geometry-related methods and fusion-related methods is mainly attributed to their effectiveness in exploiting RGB information. More specifically, the proposed method outperforms almost all the latest works by extracting extra geometric gradient information and seeking new fusion method. Different from DeepLiDAR [3], our model obtains geometry information without using additional datasets. And as a member of geometry-related type, our network uses gradient information more effectively through the proposed multi-scale gradient extractor, and thus obtains 4 mm error reduction over TWISE [59].

**Table 1:** Quantitative comparison on NYU Depth v2 dataset.

Method	RMSE↓	REL↓	*δ*_1.25_↑	*δ*_1.25^2^_↑	*δ*_1.25^3^_↑
***Bilateral*** [54]	0.479	0.084	92.4	97.6	98.9
***Zhang et al*.** [2]	0.228	0.042	97.1	99.3	99.7
***Sparse* − *to* − *Dense*** [21]	0.204	0.043	97.8	99.6	99.9
***NConv* − *CNN*** [60]	0.129	0.018	99.0	99.8	100.0
***DepthCoeff*** [1]	0.118	0.013	99.4	99.9	-
***CSPN*** [35]	0.117	0.016	99.2	99.9	100.0
***DeepLiDAR*** [3]	0.115	0.022	99.3	99.9	100.0
***GAENet*** [58]	0.114	0.018	99.3	99.9	100.0
***Depth* − *Normal*** [4]	0.112	0.018	99.5	99.9	100.0
***FCFRNet*** [20]	0.106	0.015	99.5	99.9	100.0
***ACMNet*** [17]	0.105	0.015	99.4	99.9	100.0
***PRNet*** [57]	0.104	0.014	99.4	99.9	100.0
***GuideNet*** [16]	0.101	0.015	99.5	99.9	100.0
***TWISE*** [59]	0.097	0.013	99.6	99.9	100.0
***SPNet* (*Ours*)**	0.093	0.013	99.6	99.9	100.0

#### Qualitative comparison

**Fig 5:**
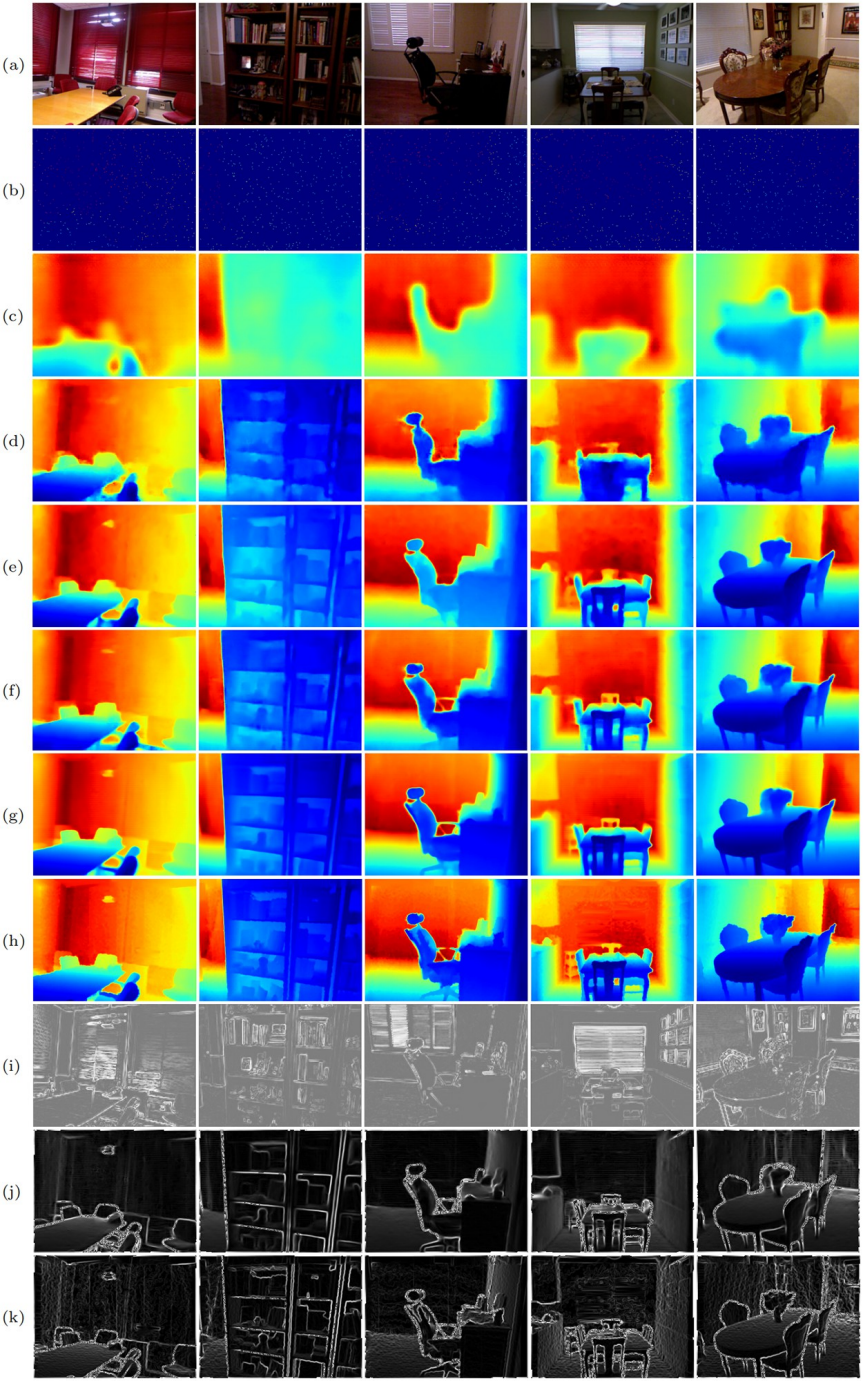
Qualitative comparisons of different methods on NYU-depth V2. (a) RGB, (b) sparse depth, (c) Sparse-to-Dense [21], (d) NConv-CNN [60], (e) CSPN [35], (f) GAENet [58], (g) SPNet (ours), (h) GT, (i) gradients from RGB images, (j) gradients from predicted depth, (k) gradients from GT depth.

For qualitative comparison, we select some examples from four representative methods, including Sparse-to-Dense [21], NConv-CNN [60], CSPN [35], and GAENet [58]. As shown in Fig 5, compared to other algorithms (Fig 5(c)–5(f)), our method (Fig 5(g)) exhibits a performance superiority in recovering structure details by combining RGB gradient (Fig 5(i)) and depth gradient (Fig 5(j) and 5(k)). It can be observed that our method better preserves small and thin structures, such as the chair handles, the table and chair legs, and the bookcase dividers. Moreover, our method restores sharper depth boundaries, and thus alleviates the depth value ambiguity problem.

### 4.3 Comparisons with state-of-the-arts on KITTI DC dataset

#### KITTI DC dataset

We further conduct experiments on the KITTI DC benchmark. The KITTI DC benchmark [55] is an outdoor dataset, which is composed of street views in real-world. Each sparse LiDAR map (around 5% valid pixels) has a corresponding ground-truth depth map (about 16% valid pixels) and an aligned RGB image. The dataset contains 86,898 frames for training, 1K frames that officially selected for validation and 1K frames for testing. The images in training set have a resolution of 1242×357, while the validation and test sets have a resolution of 1216×352. Since there are few valid pixels at the top of depth maps, we crop the bottom center of all input images to 1216×224 for training and testing.

#### Quantitative comparison

The quantitative results on validation set of KITTI DC benchmark is shown in Table 2. Experiments show that our method can well generalize to outdoor scenes. Note that in this experiment, we do not use the proposed gradient MAE loss, because the ground-truth depth maps of KITTI DC are too sparse to obtain precise ground-truth gradient images. However, our approach still outperforms gradient-related work [45] by a large margin (e.g., the RMSE error reduction over [45] is 138mm).

**Table 2:** Quantitative comparison on KITTI DC validation set.

Method	RMSE↓	MAE↓	iRMSE↓	iMAE↓
***DC* − 3*co*** [1]	1011.3	215.04	2.50	0.94
***DesNet*** [43]	938.45	266.24	2.95	1.13
***hwang* *et al*.** [45]	928	245.5	-	-
***TWISE*** [59]	879.40	193.40	2.19	0.81
***Sparse* − *to* − *Dense* (*gd*)** [19]	878.56	260.90	3.25	1.34
***NConv* − *CNN*** [60]	870.82	233.25	2.75	1.03
***PRR*** [57]	867.12	204.68	2.17	0.85
***GAENet*** [58]	813.83	245.08	2.66	1.23
***Depth* − *Normal*** [4]	811.07	236.67	2.45	1.11
***RGB guid*&*certainty*** [10]	802	214	-	-
***MultiStack*** [40]	798.80	223.40	2.57	1.0
***SPNet* (*Ours*)**	789.93	211.02	2.20	0.90

#### Qualitative comparison

**Fig 6:**
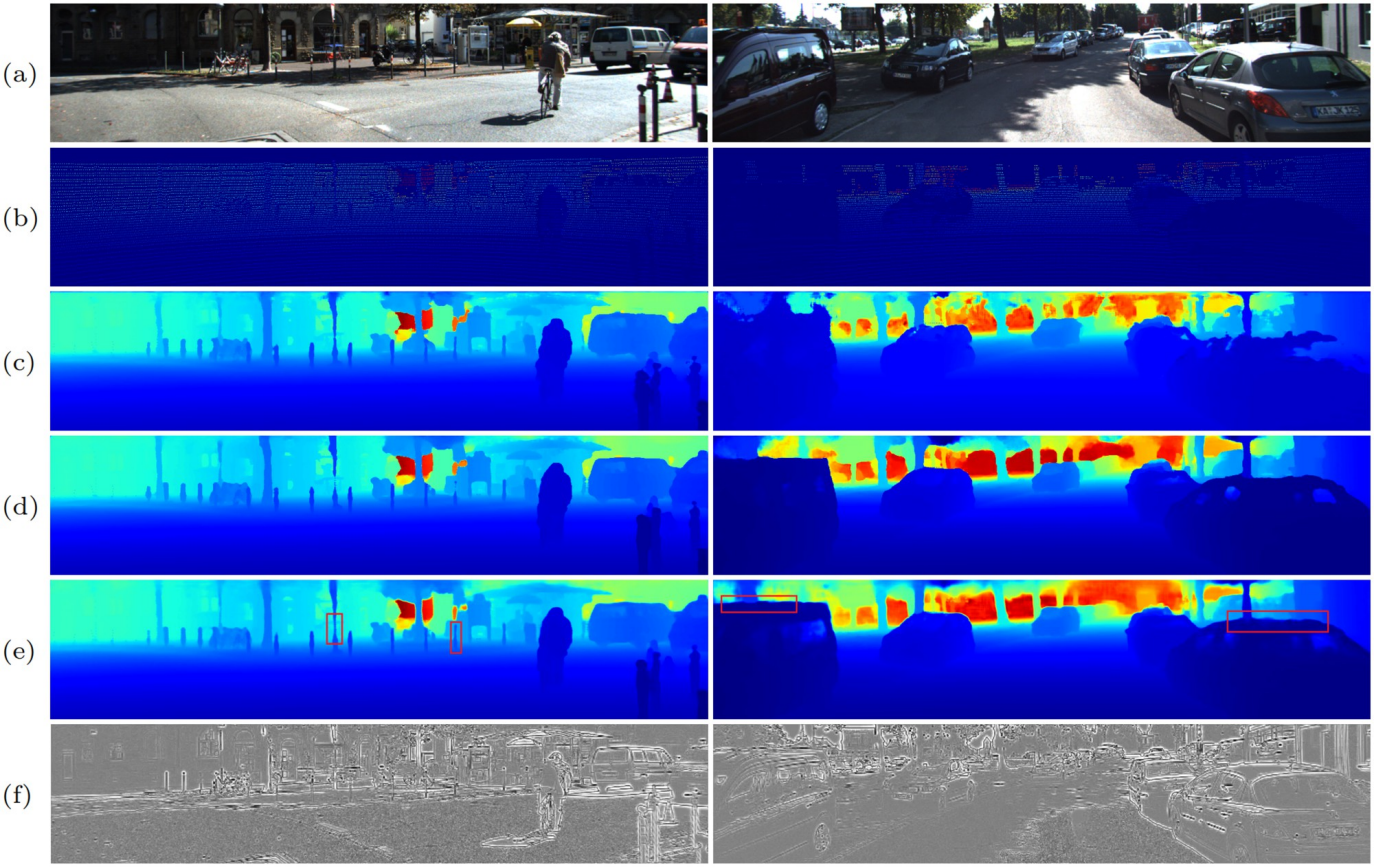
Qualitative comparisons on KITTI DC validation set. (a) RGB, (b) sparse depth, (c) NConv-CNN [60], (d) TWISE [59], (e) SPNet (ours), (f) gradients from RGB images.

Fig 6 shows some visual results of our SPNet and two state-of-the-art works [59, 60]. NConv-CNN [60] (Fig 6(c)) usually suffers from severe structural distortion (e.g. the car shape) and disappearance of tiny structure (e.g. the signage poles). TWISE [59] (Fig 6(d)) generates detailed structures, but they still have obvious boundary value errors and disappearance of tiny structure. Benefiting from the proposed multi-scale gradient extractor and multi-level feature fusion module, our method (Fig 6(e)) can preserve more geometric structures, especially on tiny objects and boundary regions. For example, our prediction produces more complete structures of the thin iron pillars and recovers sharper boundaries in the regions highlighted in the second column of Fig 6, while other methods only produce partial structures. We also illustrate the gradient images (Fig 6(f)) generated by our multi-scale gradient extractor. It can be observed that the gradient images contain rich structural information which is helpful in recovering accurate depth, as well as restraining redundant textures inside the objects.

### 4.4 Ablation study

To evaluate the effects of each component in our network, a series of ablation experiments are conducted on NYUv2 test dataset. The baseline is a single UNet network. There are three key contributions in our proposed model: the multi-scale gradient extractor, the gradient MAE loss and the multi-level feature fusion module. By combining the baseline with these three components, we can get different model variants, namely, models A-F. The quantitative results of these variants are provided in Table 3. Specifically, all of model A, model B and model C achieve some performance improvement, which reflects the individual effectiveness of these three components. Model D, model E and model F contain different combinations of these three components. Their further RMSE reductions over models A-C confirm the complementary effects of the three components. And the model *F* that with three components achieves the best performance.

**Table 3:** The effectiveness of different components.

Model	MSGE	*L*_*GMAE*_	MFFM	RMSE↓	REL↓	*δ*_1.25_ ↑	*δ*_1.25^2^_ ↑	*δ*_1.25^3^_ ↑
*baseline*				0.102	0.016	99.5	99.9	100.0
*model A*			✓	0.100	0.014	99.5	99.9	100.0
*model B*		✓		0.097	0.015	97.5	99.9	100.0
*model C*	✓			0.100	0.015	99.5	99.9	100.0
*model D*	✓	✓		0.095	0.014	99.6	99.9	100.0
*model E*	✓		✓	0.095	0.014	99.5	99.9	100.0
*model F*	✓	✓	✓	0.093	0.013	99.6	99.9	100.0

#### Analysis of multi-scale gradient extractor

**Fig 7:**
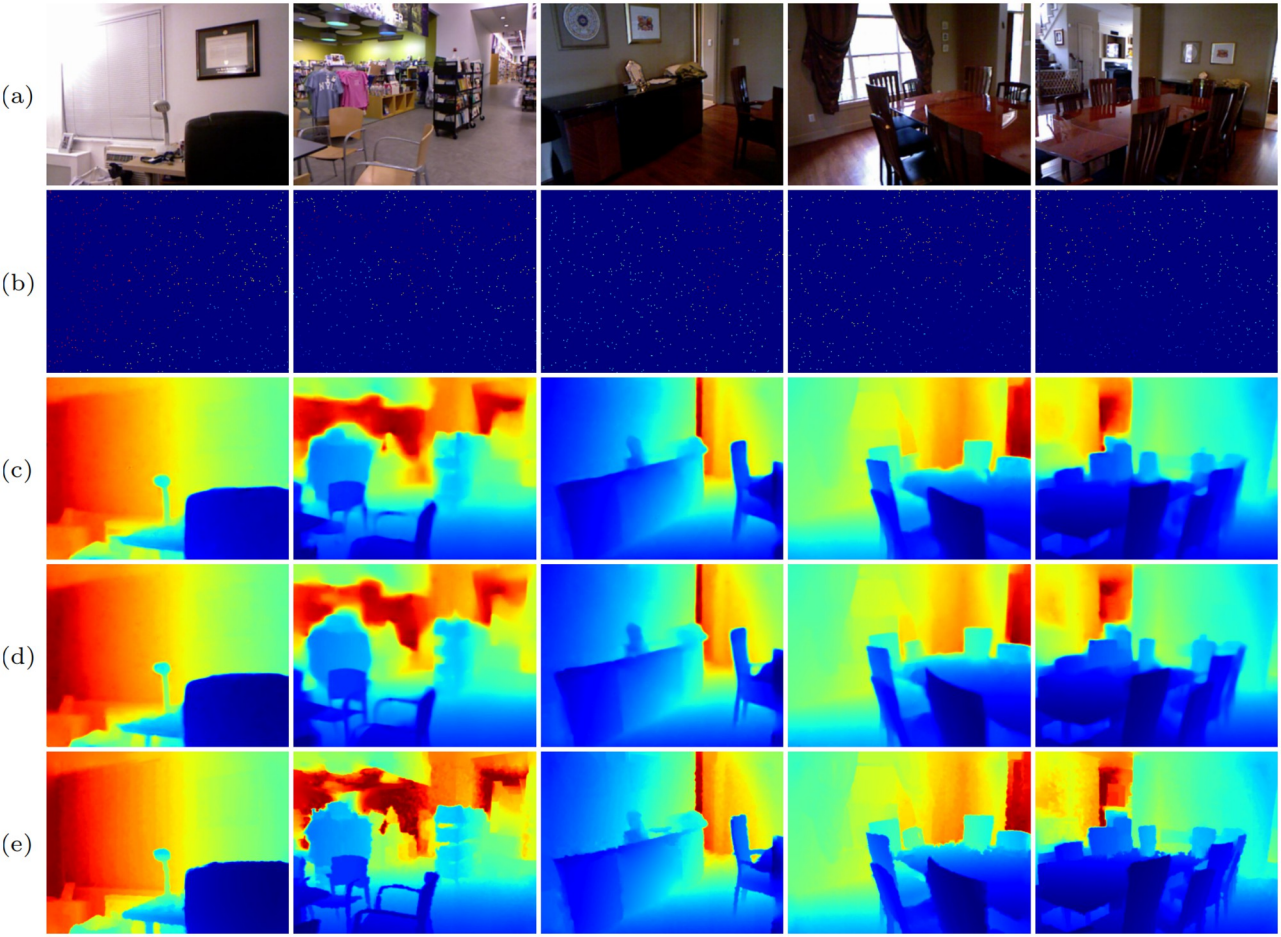
The effectiveness of multi-scale gradient extractor. (a) RGB, (b) sparse depth, (c) baseline, (d) model C, (e) GT.

To verify the effectiveness of the proposed MSGE, we add it into the baseline, obtaining model C, and then intuitively observe the visual performance. As shown in Fig 7, compared to baseline model (Fig 7 (c)), model C (Fig 7 (d)) recovers more structural details and better alleviates the depth value ambiguity problem. This indicates that MSGE can not only extract useful structural information from RGB images, but also adaptively decrease the influence of intensity variations (caused by over-exposure and under-exposure) on completion.

**Fig 8:**
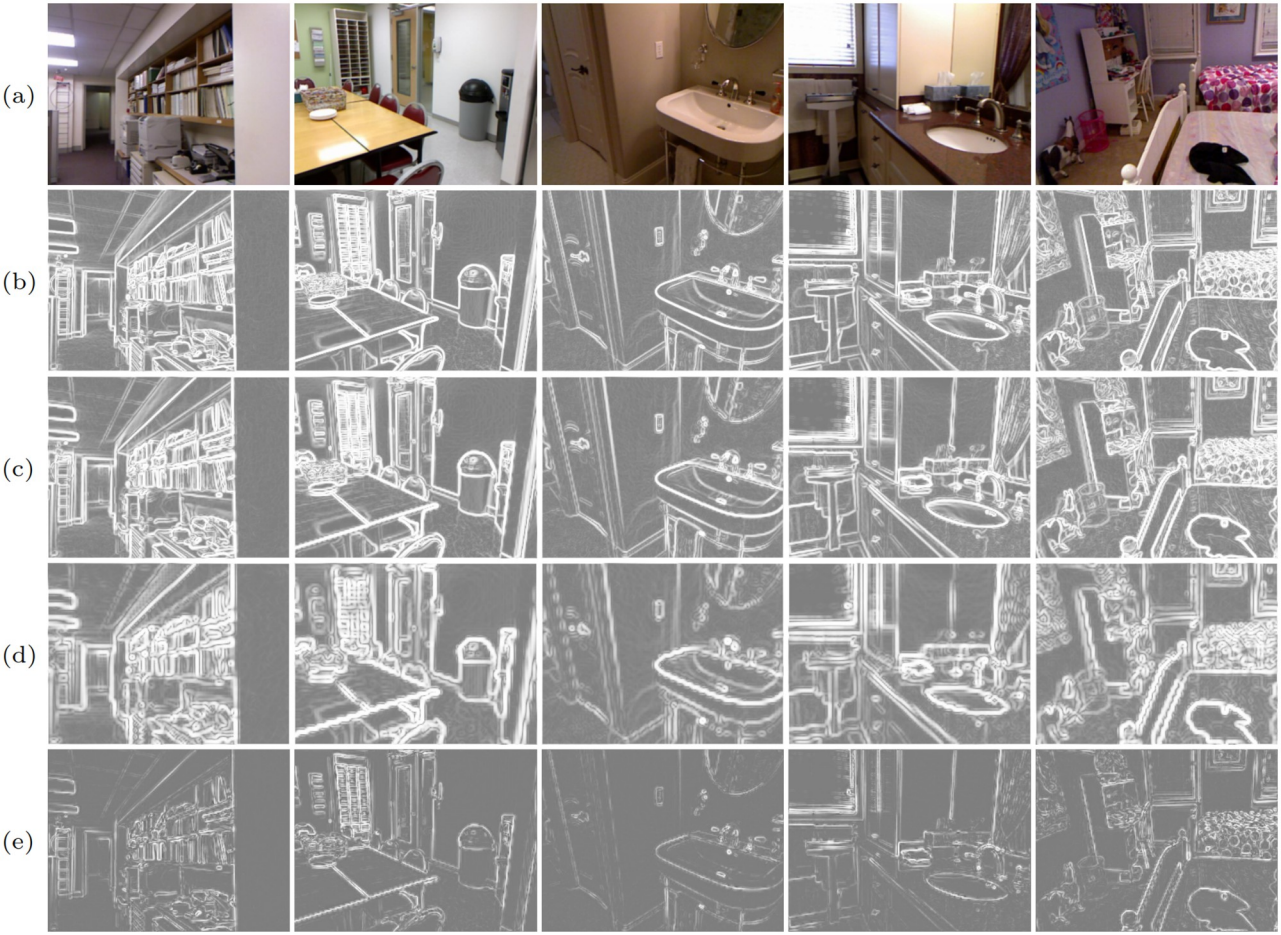
The gradient image analysis of MSGE. (a) RGB, (b) no sampling, (c) 2× sampling, (d) 4× sampling, (e) final gradient images.

It is not difficult to extract gradient images from RGB images. But how to make the network extract more useful gradients remains a challenging problem. The small-scale gradient images (Fig 8 (b)) contain both the boundary information of small-size objects and the texture information inside the large-size objects. The texture gradients should be discarded. Otherwise, they will bring explicit texture transferring artifacts. The large-scale gradient images (Fig 8 (d)) can eliminate the redundant texture inside large-size objects, but will blur or even eliminate the boundaries of small-size objects. To make full use of different scale gradient images and integrate their advantages, we extract multi-scale gradient images and then adaptively combine their information. Thus, our MSGE can not only retain small-size object boundaries, but also eliminate redundant textures in the large-size objects, as shown in Fig 8 (e).

#### The effectiveness of gradient MAE loss (*L*_*GMAE*_)

**Fig 9:**
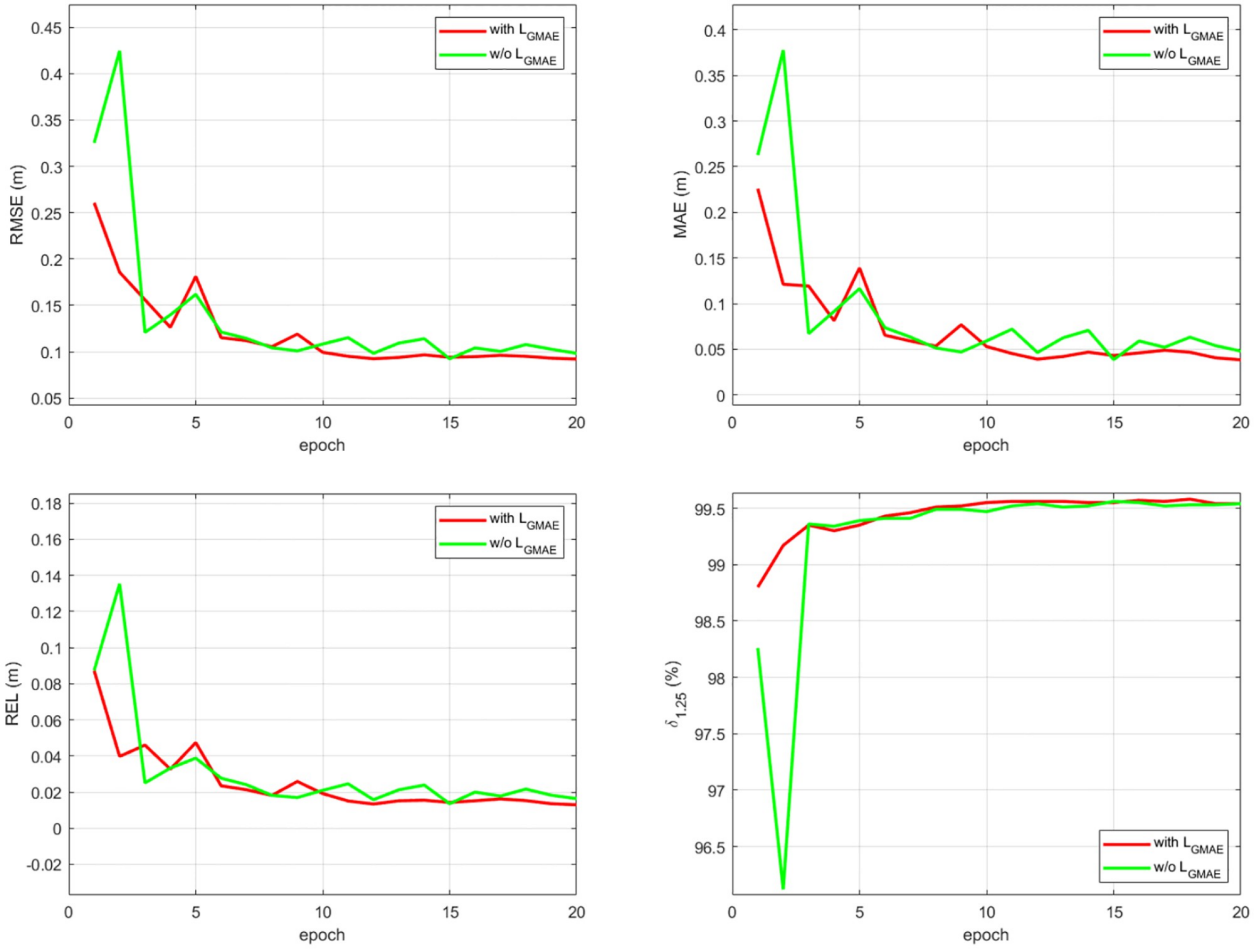
Convergence comparison between with and w/o *L*_*GMAE*_.

**Fig 10:**
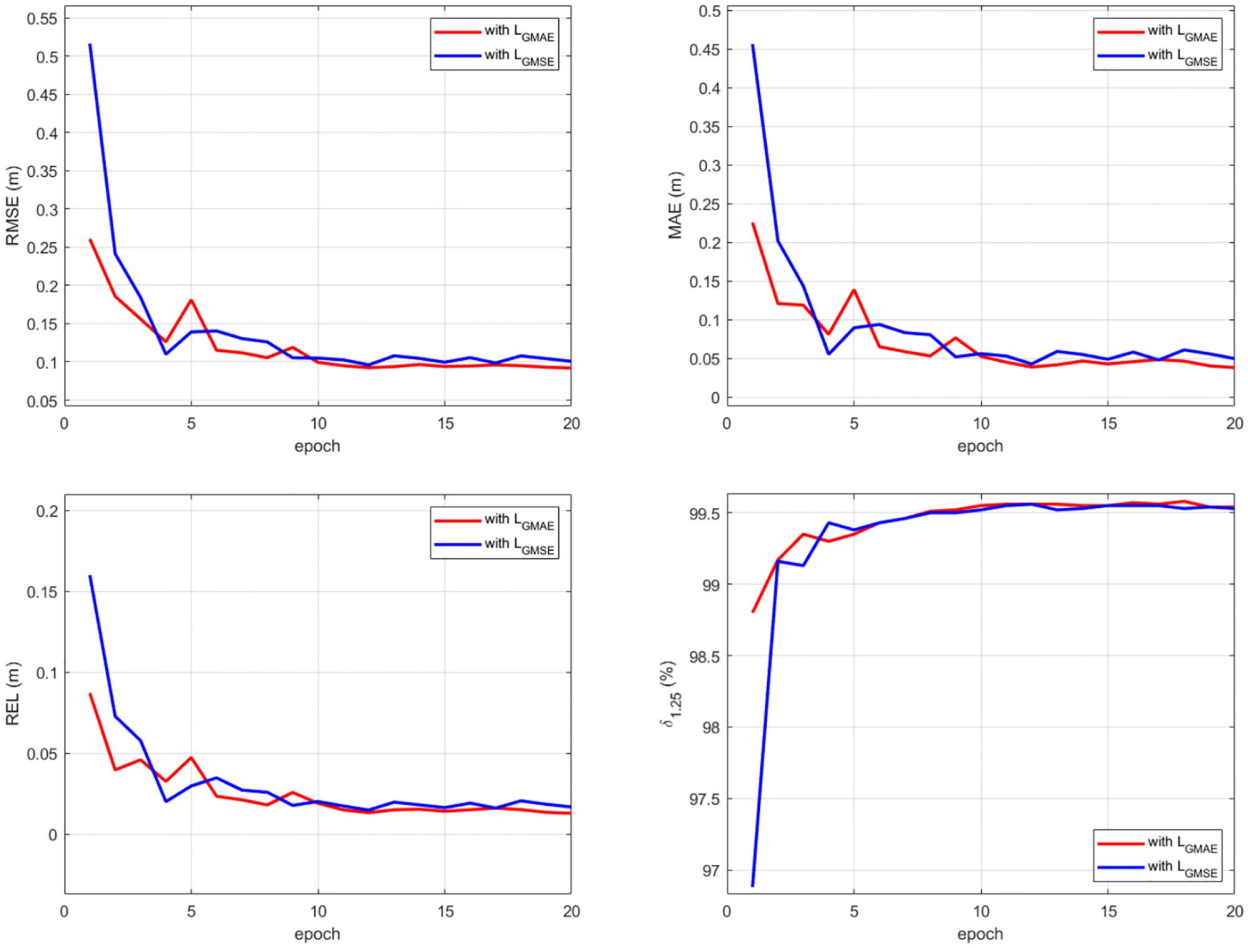
Convergence comparison between *L*_*GMAE*_ and *L*_*GMSE*_.

To evaluate the effect of *L*_*GMAE*_, we train the same model with different forms of loss functions. Firstly, we train the model with and without *L*_*GMAE*_, respectively. From Fig 9, we can see that the model with *L*_*GMAE*_ achieves a much more stable convergence, and finally reaches its convergent state with lower training errors than the model w/o *L*_*GMAE*_. Further, we compare the performance of *L*_*GMAE*_ with that of gradient MSE loss (*L*_*GMSE*_), which has been used in hwang et al. [45] and DeepDNet [50]. As shown in Fig 10, *L*_*GMAE*_ still have a higher performance than *L*_*GMSE*_. The *L*_*GMAE*_ encourages consistency of depth gradients. Since the depth gradient images can provide extra geometric constraints, the model trained with *L*_*GMAE*_ can better restore tiny structures and enhance boundary sharpness.

#### Analysis of multi-level feature fusion module (MFFM)

The simple element-wise addition and concatenation are commonly used to fuse the encoder and decoder features. As shown in Table 3, the performance gap between model A and baseline proves that the MFFM can improve the efficiency of feature fusion through the proposed enhancement attention and complementation attention. This performance improvement is mainly because the MFFM considers the distinction between two modalities and selectively incorporates the spatial details of RGB modality into the depth modality.

## 5 Conclusion

Aiming at the problems of structure lack and modal distinction in depth completion, we propose a structure preserving network in this paper. We design a multi-scale gradient extractor to efficiently capture gradient images from RGB images through the proposed semi-fixed depthwise separable convolution. This extractor can retain useful gradients and provide additional structural information for network. Thus, more structure will be preserved and more accurate depth completion results can be achieved. Simultaneously, we adopt a stable gradient MAE loss to encourage consistency of depth gradients, which can make the model pay more attention to geometric structures and object boundaries. Besides, we propose a multi-level feature fusion module to adaptively fuse spatial details (from low-level encoder features) and semantic information (from high-level decoder features) by replacing the traditional sum or concatenation operation. It has been experimentally proved that our method is effective on both indoor (NYUv2) and outdoor (KITTI) datasets. In future work, we plan to investigate how to apply gradient MAE loss to datasets that GT depth is particularly sparse.
